# A Malaysian consensus recommendation for the prevention of influenza in older persons

**DOI:** 10.1186/s12879-022-07920-3

**Published:** 2022-12-15

**Authors:** Maw-Pin Tan, Zamberi Sekawi, Roslina Abdul Manap, Rizah Mazzuin Razali, Hazlina Mahadzir, Nordiana Nordin, Kar-Chai Koh, Pui-Li Wong, Kejal Hasmuk, Noor Harzana Harrun, Siti Aisah Mokhtar

**Affiliations:** 1 Ageing and Age-Associated Disorders Research Group, Department of Medicine, Faculty of Medicine, Universiti Malaya, Jalan Profesor DiRaja Ungku Aziz, 50603 Kuala Lumpur, Wilayah Persekutuan Kuala Lumpur Malaysia; 2 Universiti Putra Malaysia, Serdang, Selangor Malaysia; 3 National University of Malaysia, Cheras, Selangor Malaysia; 4 Hospital Kuala Lumpur, Kuala Lumpur, Wilayah Persekutuan Kuala Lumpur Malaysia; 5 Hospital Canselor Tuanku Muhriz, Cheras, Wilayah Persekutuan Kuala Lumpur Malaysia; 6 KPJ Damansara Specialist Hospital, Petaling Jaya, Selangor Malaysia; 7 Poliklinik Kepong Baru, Kepong, Wilayah Persekutuan Kuala Lumpur Malaysia; 8 Universiti Malaya, Kuala Lumpur, Wilayah Persekutuan Kuala Lumpur Malaysia; 9 University Malaya Medical Centre, Kuala Lumpur, Wilayah Persekutuan Kuala Lumpur Malaysia; 10 Pandamaran Health Clinic, Pelabuhan Klang, Selangor Malaysia

**Keywords:** Human influenza, Older person, Aged, Prevention, Delphi technique, Malaysia

## Abstract

**Background:**

Older persons are at high-risk of developing severe complications from influenza. This consensus statement was developed to provide guidance on appropriate influenza prevention strategies relevant to the Malaysian healthcare setting.

**Methods:**

Under the initiative of the Malaysian Influenza Working Group (MIWG), a panel comprising 11 multi-speciality physicians was convened to develop a consensus statement. Using a modified Delphi process, the panellists reviewed published evidence on various influenza management interventions and synthesised 10 recommendations for the prevention of influenza among the aged population via group discussions and a blinded rating exercise.

**Results:**

Overall, annual influenza vaccination is recommended for individuals aged ≥ 60 years, particularly those with specific medical conditions or residing in aged care facilities (ACFs). There is no preference for a particular vaccine type in this target population. Antiviral agents can be given for post-exposure chemoprophylaxis or when vaccine contraindication exists. Infection control measures should serve as adjuncts to prevent the spread of influenza, especially during Hajj.

**Conclusion:**

This consensus statement presents 10 evidence-based recommendations that can be adopted by healthcare providers to prevent influenza among the aged population in Malaysia. It could also serve as a basis for health policy planning in other lower- and middle-income countries.

## Introduction

Influenza (commonly known as flu) is a respiratory infectious disease caused by the influenza virus that mainly infects the nose, throat, and sometimes lungs [1]. Most people recover from uncomplicated influenza but certain populations, particularly those aged 60 years and above, are considered at higher risk of developing influenza-related complications that may result in severe disease and death [2, 3]. In 2017, the World Health Organization (WHO) indicated that 290,000 to 650,000 annual global deaths were associated with influenza [4]. A recent study estimated that the global mean annual influenza-associated excess respiratory mortality rate ranged from 0.1 to 6.4 per 100,000 individuals among people aged ≤ 64 years, 2.9–44.0 per 100,000 individuals among people aged 65–74 years, and 17.9–224.5 per 100,000 individuals among people aged ≥ 75 years [5]. These estimates suggest a greater annual burden of influenza than the figures reported by WHO in 2017, underscoring the impact that influenza has on healthcare systems worldwide.

Older persons typically suffer the most severe health effects of influenza as they have an ageing immune system (immunosenescence) and more medical comorbidities [6]. According to the US Centers for Disease Control and Prevention (CDC), approximately 90% of influenza-related deaths and up to 70% of influenza-related hospitalisations have occurred among older persons aged ≥ 65 years [7]. It has been reported that older persons in Hong Kong, specifically those aged ≥ 65 years, are about 14 times more likely to die from influenza compared with adults in the 40–65 years age group [8]. In Singapore, influenza-related deaths were 11.3 times higher among older persons than in the general population [9]. From 2010 to 2017, 16.3% of all pneumonia and influenza hospitalisations in Singapore were estimated to be attributed to influenza, with a higher excess rate estimated for individuals aged ≥ 65 years (338.0 per 100,000 person-years) [10]. In Malaysia, influenza has a disproportionately higher impact on older persons, with 28.3% of patients aged ≥ 65 years experiencing hospitalisation, intensive care unit admission, or death within a year owing to influenza-related illnesses or complications compared with 9.6% of patients in the 25–64 years age group [11]. Therefore, the prevention of influenza in this high-risk group is of great importance.

In 2020, there were 2.2 million (6.8%) people in Malaysia aged ≥ 65 years and this number is projected to reach six million by 2040 [12, 13]. To address the growing need of having a localised guideline on the prevention of influenza among the aged population in Malaysia, a consensus panel was convened to develop a consensus statement that lists a set of evidence-based recommendations, aimed at raising awareness among healthcare providers and caregivers about the importance of influenza prevention among older persons, and providing guidance on appropriate influenza prevention strategies that are relevant to the Malaysian healthcare setting.

## Methods

The MIWG was formed by the Malaysian Society of Infectious Diseases and Chemotherapy (MSIDC), comprising an interdisciplinary group of experts from the fields of paediatrics, public health, infectious diseases, virology, respiratory medicine, and geriatrics, to advocate for influenza prevention in the general population in Malaysia. In 2020, a sub-committee (senior section) of MIWG was formed as an initiative between MSIDC and the Malaysian Society of Geriatric Medicine to specifically lobby for influenza vaccination in older adults. The group recognised that having appropriate guidelines for influenza prevention would strengthen the call for reducing the impact of influenza on the aged population, among policymakers, healthcare practitioners and the public. The MIWG subsequently invited 11 physicians (five consultant geriatricians, a senior consultant medical microbiologist, a senior consultant respiratory physician, a general practitioner, an infectious diseases physician, a family medicine specialist, and a public health medicine specialist) from public academic centres, public and private primary healthcare centres, as well as a private clinic to be members of the consensus panel.

A literature search of the MEDLINE database was conducted using the PubMed search engine to identify influenza-related studies—involving older persons (defined as individuals aged ≥ 60 years based on the guidelines provided by the Malaysian Ministry of Women, Family and Community Development and the Ministry of Health) [14, 15], residents and staff of ACFs, older persons with comorbidities and/or immune suppression, as well as older Hajj pilgrims—that have been published over a 15-year period (2006–2021). The following medical subject headings (MeSHs) and subheading were used as the search terms: Influenza; aged; prevention and control; and diagnosis. The inputted search syntax was: “(((“Influenza, Human“[MeSH]) AND (“Aged“[MeSH])) AND (“prevention and control“[Subheading])) AND (“Diagnosis“[MeSH]); Filters: Guideline, Meta-Analysis, Randomised Controlled Trial, 2006–2021”.

A total of 152 relevant articles were retrieved from MEDLINE. In addition, recommendations of interest were also sourced from the verified websites of medical organisations and societies. Upon reviewing the pool of information generated via the said search strategy, the consensus panel drafted 22 preliminary statements that were deemed valuable in guiding the development of the influenza prevention consensus document. These statements were subsequently subjected to a modified Delphi process where the value of various interventions in preventing influenza was scrutinised and debated to derive a list of evidence-based recommendations that would meet the objectives of this study.

Specifically, the modified Delphi technique used in this study consisted of two virtual expert meetings and one blinded rating exercise.

### First expert meeting

A set of 22 preliminary statements describing various interventions for the prevention of influenza was presented for discussion. Having reviewed the statements and their corresponding supporting evidence, panellists indicated their position on the statements by openly voting for or against their inclusion in the proposed consensus document. Only statements with ≥ 70% of positive responses were shortlisted for the subsequent blinded Delphi rating exercise. During this meeting, panellists also amended the shortlisted statements for accuracy and clarity. A total of 13 statements were eventually shortlisted for the next step.

### Blinded Delphi rating

The 13 shortlisted statements from the first expert meeting were subjected to a blinded Delphi rating process. Guided by scientific evidence, members of the consensus panel independently and anonymously rated each statement based on a 5-point Likert scale (‘strongly disagree’, ‘disagree’, ‘neutral’, ‘agree’, ‘strongly agree’). For each statement, the experts could also suggest minor amendments to the original phrasing, which was discussed in the second expert meeting. A consensus was defined as a combined ‘strongly agree’ and ‘agree’ acceptance rate of ≥ 70% [16, 17]. A total of 12 statements achieved consensus after the rating process. One statement did not achieve the minimum level of support required and was further discussed at the next meeting.

### Second expert meeting

Results of the blinded Delphi rating exercise were compiled and shared with members of the MIWG during a second virtual expert meeting. During this meeting, the panellists reviewed the reported results and appropriate revisions were made to the accepted consensus statements. At the end of the meeting, a total of 10 consensus statements were finalised as the official recommendations of the MIWG for the prevention of influenza in the aged population.

## Results and discussion

### Recommendation 1: all persons aged 60 years and above who do not have contraindications should receive an influenza vaccine annually

Older persons represent 90% of influenza-related deaths and 50–70% of influenza-related hospitalisations [7]. A study that examined the effectiveness of influenza vaccine in seniors over an extended duration found that influenza vaccination was associated with a 27% reduction in the risk of hospitalisation due to influenza or pneumonia, and a 48% reduction in the risk of death [18]. Separately, a modelling study documented that influenza vaccination in Europe averted an average of 1.6–2.1 million cases of influenza, 45,300–65,600 hospitalisations, and 25,200–37,200 deaths each year [19].

A cost-benefit analysis also showed that vaccination would result in net savings of Malaysian Ringgit (MYR) 313 for each vaccinated older person and net savings of MYR 3565 per life-year gained [20]. While influenza vaccines have a good safety profile in general, their use may be limited by certain contraindications [1]. For example, people who are allergic to any components, adjuvants, or trace ingredients in influenza vaccines (e.g., neomycin, formaldehyde, or Triton X-100) and those who have prior experience of anaphylaxis after vaccination should not be vaccinated [21, 22]. For older persons with egg allergy, influenza vaccines produced using nonegg-based techniques (e.g., trivalent and quadrivalent recombinant influenza vaccines) can be considered for administration, though available published reviews have reported that severe allergic reactions to currently available egg-based influenza vaccines in individuals with egg allergy are unlikely [23]. Nonetheless, it is recommended that older persons who have a history of severe allergic reactions to eggs be vaccinated in a medical setting under the supervision of a healthcare worker who is able to identify and manage severe allergic conditions [24].

### Recommendation 2: older persons with comorbidities should be prioritised for annual influenza vaccination

Older persons with comorbidities, including chronic lung diseases (e.g., chronic obstructive pulmonary disease, asthma, bronchiectasis, and lung fibrosis), heart diseases (e.g., heart failure and coronary artery disease), kidney and liver disorders, endocrine (e.g., diabetes melitus), and neurological disorders are at higher risk for influenza-related complications [25, 26]. In Malaysia, older persons are more likely to have ≥ 2 comorbidities with advanced age being an independent predictor of poor health outcomes [11]. This population should be prioritised for annual influenza vaccination, especially during periods of vaccine shortage. A randomised, double-blind, multicentre, active-controlled trial found that vaccination against influenza was 22.1% effective in preventing influenza among older persons with at least one high-risk comorbid condition and 23.6% effective among those with at least two high-risk comorbid conditions [27].

Moreover, a prospective, randomised, open with blinded endpoint study involving 439 patients who had been admitted to hospital for acute coronary syndrome (ACS) reported that patients who received influenza vaccination had a lower rate of developing major adverse cardiovascular events (including death and hospitalisation due to ACS, heart failure, or stroke) than those who did not (9.5% vs. 19.3%; unadjusted hazard ratio, 0.70 [0.57–0.86]; p = 0.004) [28].

### Recommendation 3: older persons should receive any available influenza vaccine approved for use in their age group

Currently, more than one licensed influenza vaccine has been approved for use in older persons. They are available as trivalent or quadrivalent, cell-cultured or egg-based, standard-dose or high-dose, and adjuvanted or nonadjuvanted vaccines. Regardless of the vaccine type, antibodies typically develop in the body approximately two weeks after vaccination [29]. Presently, three influenza vaccines are preferentially recommended for older persons: Fluzone high-dose quadrivalent, Flublok quadrivalent recombinant, and Fluad quadrivalent adjuvanted influenza vaccines [30]. If none of the three influenza vaccines is available, older persons who have no contraindication to influenza vaccination should receive any registered and age-appropriate influenza vaccine [30]. Generally, the availability of influenza vaccines can vary from year to year and from country to country [31]; clinicians in Malaysia should administer whichever formulation that is available locally to achieve the highest possible vaccine coverage among aged individuals (Table 1).

**Table 1:** Influenza vaccines currently available in Malaysia (as of June 2022)

Influenza vaccine	Vaccine type
Fluarix tetra	Egg-based
Influvac tetra	Egg-based
FluQuadri quadrivalent	Egg-based
Vaxigrip tetra	Egg-based
SKYCellflu quadrivalent	Cell-based

### Recommendation 4: post-exposure prophylaxis with antiviral agents can be considered within 48 h for preventing the transmission of influenza in older persons

Post-exposure prophylaxis (PEP) with antiviral medications can be prescribed to older persons to prevent the transmission of influenza and should be given within 48 h of exposure to a person with influenza for 7 days after the last known exposure [2]. PEP is particularly important for those who cannot receive vaccination owing to contraindications or if they are severely immunocompromised [2]. A randomised, double-blind study assessing the effectiveness of a neuraminidase inhibitor, oseltamivir, as PEP among close contacts of individuals with influenza showed that the risk of developing influenza was reduced by 89% compared with those who received a placebo [32].

PEP with another neuraminidase inhibitor, zanamivir, protected close contacts with similarly high prophylactic efficacy, ranging from 82 to 84% [33, 34]. In 2020, a selective inhibitor of influenza cap-dependent endonuclease (i.e., baloxavir) was approved by the US Food and Drug Administration (FDA) for influenza PEP [35]. It has been demonstrated that close contacts of people with influenza who were given baloxavir were 86% less likely to contract influenza after exposure to an infected person compared with those who received a placebo (1.9% [7 of 374] vs. 13.6% [51 of 375]; adjusted risk ratio, 0.14; 95% confidence interval [CI], 0.06–0.30; p < 0.001) [36].

### Recommendation 5: antiviral agents should be given as post-exposure chemoprophylaxis as soon as possible to all non-ill residents during influenza outbreaks in ACFs

An influenza outbreak at an ACF can be suspected when there is a resident with laboratory-confirmed influenza and another resident at the same facility develops influenza-like illness (ILI) within 72 h [37]. When an influenza outbreak is recognised in an ACF, chemoprophylaxis should be administered immediately to all non-ill residents, regardless of their influenza vaccination status, to prevent the spread of the virus. Oseltamivir has been shown to be 92% effective in preventing influenza in residents of ACFs exposed to influenza [38], while zanamivir was reportedly effective in controlling at least five influenza outbreaks in ACFs [39–43]. To effectively control influenza outbreaks, chemoprophylaxis should be taken for a minimum of two weeks and continued for seven days after the last known case of influenza was identified [2].

### Recommendation 6: immunocompromised older persons should receive inactivated influenza vaccine annually

Immunocompromised patients are at high-risk of contracting influenza and have significant influenza-associated excess mortality rates [44]. Annual inactivated influenza vaccination is widely recommended for this group, including those with primary immune deficiencies related to inherited diseases that affect the immune system, as well as those with secondary immune deficiencies resulting from human immunodeficiency virus (HIV) infection or acquired immunodeficiency syndrome, solid organ or stem cell transplant, cancers associated with immune deficiency, and cancer chemotherapy [45].

A randomised controlled trial indicated that the influenza vaccine was highly effective among patients with HIV, providing a protective efficacy of 100% for vaccine recipients against laboratory-confirmed symptomatic influenza (95% CI, 73–100%) [46]. Moreover, a prospective study on the immunogenicity of inactivated influenza vaccine among patients receiving cytotoxic myelosuppressive chemotherapy for solid tumours and haematologic malignancies demonstrated that the influenza vaccine could induce an immune response similar to that seen in healthy controls [47].

### Recommendation 7: influenza vaccine, education on influenza prevention, and infection control measures should be recommended to all older Hajj and Umrah pilgrims

Influenza vaccination is currently not mandatory for Malaysian Hajj and Umrah pilgrims. In a cross-sectional study conducted amongst 394 Malaysian Hajj pilgrims in 2007, 96.4% of the study population had an ILI—out of which 91.5% experienced cough; 59.2%, fever; 79.3%, runny nose; and 57.1%, sore throat [48]. Alborzi and associates (2009) found that vaccinated pilgrims contracted the influenza virus less frequently than unvaccinated pilgrims (9.2% vs. 16.5%) [49]. A case-control study on the effectiveness of influenza vaccination among Malaysian pilgrims attending the Hajj in 2000 showed that the influenza vaccine was 77% effective in preventing clinic visits for ILI and 66% effective in reducing the use of antibiotics [50]. All older Hajj pilgrims who had not been immunised are recommended to receive an influenza vaccine, preferably no less than two weeks prior to their arrival in Saudi Arabia [51].

Nonetheless, vaccination alone is not an adequate infection control measure for pilgrims. In a study assessing the incidence rate of influenza among 115 pilgrims during the 2003 Hajj season, nine out of 30 vaccinated pilgrims (30%) still experienced breakthrough infection [52]. Therefore, the implementation of other supportive infection control measures, including the use of face masks, alcohol-based hand sanitisers, and health education, is important in mitigating the potential transmission of influenza during Hajj. Previous studies reported that the regular use of a face mask was a significant protective factor in reducing the incidence of infection [53, 54]. Al-Asmary and colleagues found that Hajj pilgrims who consistently used alcohol-based hand sanitisers had more than an eight-fold lower risk of acquiring infection compared with those who did not [55]. Similarly, Alqahtani et al. revealed that pilgrims who had received health education before travelling to Hajj were twice as likely to get vaccinated than those who had no exposure to proper education materials [56]. Hence, appropriate education of pilgrims by healthcare professionals and Hajj and Umrah travel agents is recommended to maximise the adherence to influenza prevention measures among pilgrims.

### Recommendation 8: all residents of ACFs should be offered annual influenza vaccination

Menec and colleagues reported that hospitalisation and deaths due to influenza and pneumonia were about two to three times higher among older persons living in ACFs compared with those living in the community (31.9 vs. 9.8 per 1000 population, and 61.9% vs. 30.7%, respectively) [57]. Influenza vaccination is currently one of the most optimal ways of reducing the risk of hospitalisation and death among older persons who live in ACFs [58, 59]. Among vaccinated residents, there were significantly fewer clinical cases, hospitalisations, and deaths due to influenza (256 cases among 10,739 subjects, 32 hospitalisations, and one death) compared with unvaccinated residents (694 cases among 11,723 subjects, 150 hospitalisations, and five deaths) (p ˂ 0.001) [58], thus reducing the risks by 59.7%, 76.7%, and 78.2% respectively.

The influenza vaccine has also been shown to be effective not only in reducing the incidence of ILI and influenza-related hospitalisation but also in preventing serious complications caused by influenza, such as pneumonia (17% among vaccinated vs. 27% among unvaccinated residents; p < 0.05) and deaths (5% vs. 14%; p < 0.05) in the ACF setting [59]. Based on a nonrandomised, single-blind placebo control study on the effectiveness of influenza vaccination for the prevention of ILI among residents of ACF in Malaysia, influenza vaccination reduced the risk of contracting ILI by 14–45% and showed 55–76% vaccine effectiveness in reducing the occurrence of ILI [60].

### Recommendation 9: annual influenza vaccination should be offered to all staff of ACFs

In line with WHO recommendations, annual influenza vaccination should be offered to all staff who will potentially have contact with residents of ACFs, unless contraindicated [61]. In this consensus statement, the term ‘ACFs’ encompasses long-term care facilities, nursing homes, day care centres, and residential care facilities that provide care to people who are unable to live independently owing to advanced age, physical disability, or mental disability. The term ‘staff’ includes any person employed by the ACFs, caregivers, and volunteers (e.g., doctors, nurses, cooks, laundry workers, cleaners, and security staff).

A randomised controlled trial showed that the vaccination of healthcare workers not only reduced influenza infection by 88% in vaccinated individuals but also reduced absenteeism caused by influenza illness (9.9 days per 100 vaccinated staff vs. 21.1 days per 100 unvaccinated staff) [62]. Importantly, vaccination of staff at ACFs may have beneficial effects on the aged residents. The reduction of complications afforded by vaccinating staff is equivalent to preventing five deaths, two admissions to hospital with ILI, seven general practitioner consultations for ILI, and nine cases of ILI per 100 residents [63]. Moreover, a pair-matched cluster study involving 1,059 patients in 12 geriatric medical long-term care sites also found that vaccination of staff was associated with reductions in ILI (odds ratio [OR], 0.57; 95% CI, 0.34–0.94) and total patient mortality from 17 to 10% (OR, 0.56; 95% CI, 0.40–0.80) [64].

### Recommendation 10: staff and residents in ACFs with influenza-like illnesses should be isolated and tested for influenza, and outbreak control measures instituted

To prevent influenza outbreaks in ACFs, staff and residents in ACFs with ILI should be isolated from healthy individuals in a timely manner. In general, isolation should be maintained for 7 days after the onset of symptoms or until their symptoms have resolved [65]. Meanwhile, laboratory tests, including rapid influenza diagnostic tests (RIDTs), molecular assays, and virus culture should be performed to determine if their illness is due to influenza infection [21]. As compared with molecular assays such as reverse transcription polymerase chain reaction, RIDTs have a lower and inconsistent sensitivity, ranging from 10 to 80% [66, 67]. In addition, the results of RIDTs may be affected by various factors, including the prevalence of influenza activity in the population tested [66, 67]. Typically, false-negative RIDTs results are more likely to occur when the influenza prevalence is high in the community [67]. However, RIDTs have a high specificity (90–95%); thus, positive RIDTs results can rule in influenza infection but negative RIDTs results do not exclude influenza infection in individuals with symptoms suggestive of influenza [66, 67]. Confirmatory testing using molecular assay is recommended when negative RIDTs results are obtained for a symptomatic staff or resident; antiviral treatment should be initiated if clinically indicated and should not be delayed while influenza testing results are pending [2]. Standard and droplet precautions should also be implemented immediately when two cases of laboratory-confirmed influenza affecting staff or residents of the same facility are identified within 72 h (Table 2) [2, 68].

**Table 2:** Essential components of standard and droplet precautions

*Standard precautions*	
• Hand hygiene • Personal protective equipment (PPE) • Cough etiquette • Patient placement • Patient-care equipment and instrument/devices	• Care for the environment • Textiles and laundry • Safe injection practices • Infection control practices for special lumbar puncture procedures • Worker safety
*Droplet precautions*	
• Use of PPE • Patient transport	• Patient-care equipment and instrument/devices

Detailed information can be found at https://www.cdc.gov/infectioncontrol/guidelines/isolation/index.html [Accessed June 3, 2022]

## Conclusion

This consensus statement presents 10 evidence-based recommendations that healthcare providers and caregivers can adopt to prevent influenza among the aged population in Malaysia. It can be used as a fundamental guide to facilitate the planning and execution of influenza prevention strategies across various healthcare settings and environment. These recommendations may also help to inform policymaking decisions in other lower- to middle-income countries.

## Data Availability

Data sharing is not applicable to this article as all data generated and analysed during the current study are included in this article.
